# Cutoff Scanning Matrix (CSM): structural classification and function prediction by protein inter-residue distance patterns

**DOI:** 10.1186/1471-2164-12-S4-S12

**Published:** 2011-12-22

**Authors:** Douglas EV Pires, Raquel C de Melo-Minardi, Marcos A dos Santos, Carlos H da Silveira, Marcelo M Santoro, Wagner Meira

**Affiliations:** 1 Department of Biochemistry and Immunology, Universidade Federal de Minas Gerais, Belo Horizonte, 31270-901, Brazil; 2 Department of Computer Science, Universidade Federal de Minas Gerais, Belo Horizonte, 31270-901, Brazil; 3 Advanced Campus at Itabira, Universidade Federal de Itajubá, Itabira, 37500-903, Brazil

## Abstract

**Background:**

The unforgiving pace of growth of available biological data has increased the demand for efficient and scalable paradigms, models and methodologies for automatic annotation. In this paper, we present a novel structure-based protein function prediction and structural classification method: Cutoff Scanning Matrix (CSM). CSM generates feature vectors that represent distance patterns between protein residues. These feature vectors are then used as evidence for classification. Singular value decomposition is used as a preprocessing step to reduce dimensionality and noise. The aspect of protein function considered in the present work is enzyme activity. A series of experiments was performed on datasets based on Enzyme Commission (EC) numbers and mechanistically different enzyme superfamilies as well as other datasets derived from SCOP release 1.75.

**Results:**

CSM was able to achieve a precision of up to 99% after SVD preprocessing for a database derived from manually curated protein superfamilies and up to 95% for a dataset of the 950 most-populated EC numbers. Moreover, we conducted experiments to verify our ability to assign SCOP class, superfamily, family and fold to protein domains. An experiment using the whole set of domains found in last SCOP version yielded high levels of precision and recall (up to 95%). Finally, we compared our structural classification results with those in the literature to place this work into context. Our method was capable of significantly improving the recall of a previous study while preserving a compatible precision level.

**Conclusions:**

We showed that the patterns derived from CSMs could effectively be used to predict protein function and thus help with automatic function annotation. We also demonstrated that our method is effective in structural classification tasks. These facts reinforce the idea that the pattern of inter-residue distances is an important component of family structural signatures. Furthermore, singular value decomposition provided a consistent increase in precision and recall, which makes it an important preprocessing step when dealing with noisy data.

## Background

With the increasing number of genome and metagenome projects, sequence databases have grown exponentially. On the one hand, the August 2010 release of the UniprotKB/TrEMBL database [1] contains about 12,000,000 protein sequences. In the last month, more than 300,000 new sequences have been added to that repository, and about 6,000,000 entry annotations have been revised. On the other hand, the Pfam database of protein families [2] represents about 12,000 families, and about 20% of these are domains of unknown function (DUFs), revealing that state-of-the-art sequence similarity-based and even profile-based annotation methods have had limited success in assigning functions to novel proteins.

Protein structural classification databases, such as SCOP [3], also present difficulties in keeping up with the increasing number of protein structures solved and deposited in public repositories. Approximately 53% of the Protein Data Bank (PDB) [4] entries are classified by the current release of SCOP (1.75) as of April 2011, and after removing redundancy (sequence similarity at 90%), the coverage drops to about 41%. As international structural genomics initiatives have produced a huge number of structures of unknown function, attempting to automatically assign functions to these proteins is becoming even more necessary, and significant efforts have been devoted to this task [5-8].

In this context, novel paradigms, models and methodologies for automatic annotation must be investigated. Because protein structure and function are more conserved than protein sequence [9], the identification of similarities between novel sequences and known structures would greatly improve the characterization of these sequences. Fold recognition refers to identifying main structural features by the connections and positions of secondary structure elements. Conversely, according to Murzin et al. [3], structural classification is conducted at hierarchical levels (class, fold, superfamily and family) that embody evolutionary and structural relationships. In this work, we focused on structural classification, which encompasses the problem of fold recognition. Both fold recognition and structural classification are important steps toward function prediction.

Over the years, protein fold recognition has been addressed through different approaches. The authors of [10] extracted a series of features from protein sequences and used support vector machines and neural network learning methods as the base classifiers in a dataset composed of SCOP folds. Later, ensemble classifiers [11] were applied to these same feature vectors, improving the success rate. The use of a combination of sequence and structure information brought an improvement to fold recognition, as mentioned in the information retrieval approach introduced in [12].

Likewise, several efforts toward structure-based protein function prediction have been made. We can quote, for instance, the search for structural motifs [13-15] and functional residues (such as DNA [16] and metal [17] binding sites), the use of 3D templates [5] and the comparison of protein folds by structure alignments [18,19]. There have also been attempts to infer function from structure without the use of alignment algorithms, such as in enzyme classification [20,21]. Similarly, in the present work, we do not use alignment techniques or any sequence information in our method, relying only on structural grounds. A primary problem faced when dealing with protein function, as pointed out in [22], s defining the scope and function. Protein function prediction may be understood from different perspectives. It could mean the prediction of the cellular process in which a protein is involved, its enzymatic activity or even its physiological role. For instance, a protein’s enzymatic activity could be described by EC numbers, while its physiological role might be related to its subcellular localization. In this work, the aspect of protein function considered is enzyme activity. However, the study might be extended, without loss of generality, to other functional features, like the terms of the Gene Ontology (GO) [23] annotation.

Even though function cannot be directly implied from the specific fold adopted by a certain protein, structural data can be used to detect proteins with similar functions whose sequences have diverged during evolution [24]. In this context, one possible strategy is the definition of structural signatures, which are sets of features that are able to unequivocally identify a protein fold and the nature of interactions it can establish with other proteins and ligands. These feature sets are concise representations of protein structures, and we believe that their discovery and comprehension will be an important milestone in the protein function prediction field, being a step beyond sequence homology-based methods.

In this paper, we investigate a special type of feature that might be part of structural signatures: the patterns in inter-residue distances (or contacts). Proteins with different folds and functions present significant differences in the distribution of distances among residues as a consequence of the underlying interaction and packing of the atomic network, which is fundamental for defining protein folding [25]. In [26], we have used these distribution distances to compare and correlate different methodologies of protein inter-residue contacts. We found, surprisingly, that the traditional cutoff-dependent approach was a simpler, more complete and more reliable technique for contact definition than other cutoff-independent methods, such as Delaunay tessellation [27], especially when the target is the discrimination of first-order contacts. In this work, we propose using inter-residue distance patterns for protein classification.

The structural data we used are the cumulative contact distributions based on the Euclidean distances among alpha carbons, the Cutoff Scanning Matrix (CSM). The motivation for the use of this kind of information lies in the fact that proteins with different folds and functions have significantly different distributions of distances between their residues, and protein similarity is reflected in these distance distributions, information that is captured in the CSM. After generating this structural data, we apply singular value decomposition (SVD) to reduce dimensionality and noise. The processed matrix is finally submitted to different, previously described classification algorithms. Therefore, the main innovation of this work relies more on the powerful combination of the new structural feature of inter-residue contacts used as a discriminator and principal components selection by SVD rather than in the creation of a new classification method per se. Indeed, we showed our methodology to be, in general, independent of the classifiers utilized, giving even results for different classification heuristics.

Having in mind these considerations, we showed that the patterns derived from CSMs might effectively be used in automatic protein function prediction and structural classification. At first glance, in the case of enzyme function prediction, the proposed method achieved (over the superfamilies) an average precision of 98.2% (sd = 1.6) and average recall of 97.9% (sd = 2.0), using a gold-standard dataset of enzymes [28]. Using a much larger set of enzymes with their respective EC numbers (the 950 most-populated EC numbers in terms of available structures), CSM was able to achieve up to 95.1% precision and recall results. For the recall results, considering the levels of hierarchical structure of SCOP [3], we were able to accomplish an average precision of 93.5% (sd = 1.4) and average recall of 93.6% (sd = 1.4). In comparison to the state-of-the-art methods used in this context, such as that given by Jain and Hirst [29], using very similar database input (SCOP release 1.75), our methodology presented more robust and homogeneous results, with an average precision a bit below that of those authors: 90.7% versus 93.6%, but with less dispersion (sd of 3.0 versus 6.4). We had remarkably better recall results: an average of 90.7% versus 77.0%, with significantly lower dispersion (sd of 2.9 versus 18.4). Further details are discussed in the next section.

## Results and discussion

To test the ability of our method to successfully predict functions and recognize folds, we performed two sets of experiments with datasets designed for these different tasks.

For function prediction, as mentioned in the Methods section, we built one database based on manually curated protein superfamilies and another based on EC numbers to test if the present structure-based method could help in protein function annotation.

For structural classification, we performed experiments to verify our ability to assign SCOP class, superfamily, family and fold to protein domains. Furthermore, to place this work into the context of the literature, we also tested a superset of the dataset used by Jain and Hirst in [29]. As far as we know, their work presents the highest precision in protein fold recognition published thus far.

Finally, we relate some experiments that aimed to evaluate an SVD-based noise reduction strategy.

### Function prediction

In the function prediction experiments, our goal was to assess how well three different classification algorithms predict protein function according to protein EC numbers and a mechanistically diverse gold-standard database of functional family classes [28]. We used 10-fold cross validation for all the experiments.

For the dataset of the top 950 most-populated EC numbers, CSM was able to achieve 95.1% precision and recall after SVD processing using the KNN (K-Nearest Neighbors) algorithm. The four levels of the EC number were used together as the classes to train and test the classifier. Additional file 1, Figure S1 shows the variation in the performance metrics for each EC number class considered. Even though the number of proteins assigned to each EC number is very unbalanced, the majority of classes were classified properly, with high quality according to the metrics extracted.

Considering the gold-standard dataset, without SVD and using KNN, our method achieved an average precision of 94.2% (sd = 5.5) and a recall of 94.5% (sd = 5.5) (Table 1). For naive Bayes, it achieved 82.3% (sd = 13.8) precision and 79.2% (sd = 15.4) recall (Additional file 1, Table S1), and for random forest, it achieved 92.0% (sd = 6.9) precision and 91.6% (sd = 7.2) recall (Additional file 1, Table S2). We also showed that by using SVD, we may significantly improve these results, and in the worst case, we had 94.6% precision and 93.1% recall for the enolase superfamily using naive Bayes. The KNN and random forest methods were able to detect isoprenoid synthase type I with 100% precision and recall. Additionally, we performed experiments using all six superfamilies to train a single classifier. In this scenario, even with a greater number of families in the training and testing phases, we were still able to achieve up to 99.0% precision with KNN and random forest after SVD preprocessing.

**Table 1:** Function prediction performance using KNN for the gold-standard dataset

Superfamily	Before SVD		After SVD		∆Prec.	∆Rec.
	*Precision*	*Recall*	*Precision*	*Recall*		
Amidohydrolase	0.983	0.983	1.000	1.000	+1.7%	+1.7%
Crotonase	0.955	0.953	0.979	0.977	+2.4%	+2.4%
Enolase	0.876	0.853	0.971	0.967	+9.5%	+11.4%
Haloacid Dehalogenase	0.881	0.925	0.984	0.981	+10.3%	+5.6%
Isoprenoid Synthase Type I	1.000	1.000	1.000	1.000	+0.0%	+0.0%
Vicinal Oxygen Chelate	1.000	1.000	1.000	1.000	+0.0%	+0.0%
All	0.901	0.903	0.991	0.989	+9.0%	+8.6%

Prediction performance for the gold-standard dataset using KNN. The experiment was performed in an intra-superfamily fashion, and the *classes* for prediction represent the enzyme’s families. The precision and recall metrics are weighted averages. Ten-fold cross validation was employed.

### Protein structural classification

To the best of our knowledge, no test of the structural classification of very large databases, such as the entire SCOP containing about 110,000 domains, has been published. Due to SVD dimensionality reduction ability and the possibility of representing protein instances by a few significant attributes, we present a method that can efficiently handle such volume of data.

We may recognize protein folds at a 92.2% precision and 92.3% recall using KNN (Table 2). Even broad proteins categories, such as the SCOP class level, can be separated using CSM with very significant precision and recall (95.4% for both). The proposed method was able to classify proteins in the four levels of SCOP hierarchy with very high precision and recall, showing that CSM is a suitable method for fold recognition and also that CSMs are a very promising component of protein structural signatures. Additionally, we verified the impact of imposing a minimum number of entities per node of the SCOP hierarchy on the precision of the prediction. Additional file 1, Figure S2 shows an approximately linear correlation between these variables for the fold, superfamily and family levels with and without the SVD processing. This correlation was not analyzed for the class level because all of the classes have more than 100 entities.

**Table 2:** Structural classification performance using KNN for the Full-SCOP dataset

SCOP Level	Before SVD		After SVD		∆Prec.	∆Rec.
	*Precision*	*Recall*	*Precision*	*Recall*		
Class	0.927	0.926	0.954	0.954	+2.7%	+2.8%
Fold	0.868	0.869	0.922	0.923	+5.4%	+5.4%
Superfamily	0.871	0.872	0.926	0.927	+5.5%	+5.5%
Family	0.888	0.889	0.938	0.938	+5.0%	+4.9%

Prediction performance for the full-SCOP dataset using KNN. The experiment was performed for each classification level of SCOP. The precision and recall metrics are weighted averages. A 10-fold cross validation was employed.

### Performance comparison

**Figure 1:**
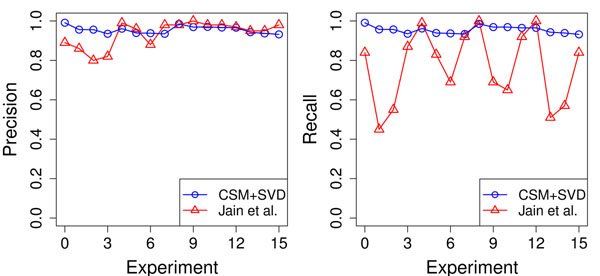
**Comparison of precision and recall.** A comparison of the prediction performance of the CSM+SVD approach and the work of Jain and colleagues in terms of precision and recall. CSM, while achieving a compatible level of precision, presents a significant improvement in recall.

In [29], the authors presented a random forest-based method to predict the SCOP class, fold, superfamily and family levels based on secondary structure element descriptors that achieved precisions of up to 99.0%. Using a similar dataset, we tried to compare our results to theirs. As far as we are concerned, this was the state-of-art method for automatic structural classification. They used a subset of SCOP database as they aimed to recognize protein folds. In our comparison of results, we were able to achieve similar precision levels but with higher recall (overcoming in up to 50.0%) in most of the cases. In only 3 of the 16 experiments, we obtained a lower recall value with our method and our F1 scores were also superior. The complete set of information regarding this experiment is available in Table 3. Figure 1 shows the performance comparison for each experiment in terms of precision and recall. CSM significantly overcomes the recall of the aforementioned study while preserving a compatible precision level. We stress that our method is not limited to small proteins. These results show that our method is not only comparable to [29] but also presents a considerable gain in terms of recall.

**Table 3:** Comparison of prediction performance

Dataset	SCOP level	CSM+SVD			Jain et al.			∆Prec.	∆Rec.
		*Prec .*	*Recall*	*F1*	*Prec .*	*Recall*	*F1*		
3SSE	Class	0.991	0.991	0.991	0.890	0.840	0.864	+10.1%	+15.1%
	Fold	0.956	0.957	0.956	0.860	0.450	0.591	+9.6%	+50.7%
	Superfamily	0.956	0.957	0.956	0.800	0.550	0.652	+15.6%	+40.7%
	Family	0.935	0.935	0.935	0.820	0.870	0.844	+11.5%	+6.5%
4SSE	Class	0.961	0.962	0.961	0.990	0.990	0.990	-2.9%	-2.8%
	Fold	0.939	0.939	0.938	0.960	0.830	0.890	-2.1%	+10.9%
	Superfamily	0.938	0.937	0.937	0.880	0.690	0.774	+5.8%	+24.7%
	Family	0.935	0.934	0.933	0.980	0.920	0.949	-4.5%	+1.4%
5SSE	Class	0.985	0.985	0.985	0.980	1.000	0.990	+0.5%	-1.5%
	Fold	0.969	0.969	0.969	1.000	0.690	0.817	-3.1%	+27.9%
	Superfamily	0.970	0.969	0.969	0.980	0.650	0.782	-1.0%	+31.9%
	Family	0.967	0.965	0.965	0.980	0.920	0.949	-1.3%	+4.5%
6SSE	Class	0.966	0.965	0.965	0.970	1.000	0.985	-0.4%	-3.5%
	Fold	0.943	0.943	0.942	0.950	0.510	0.664	-0.7%	+43.3%
	Superfamily	0.937	0.939	0.937	0.950	0.570	0.713	-1.3%	+36.9%
	Family	0.932	0.932	0.930	0.980	0.840	0.905	-4.8%	+9.2%

A comparison of prediction performance between the current study and the method introduced by [29]. The precision and recall metrics are weighted averages. This result comprises a 10-fold cross validation in KNN.

### Noise reduction strategy

As we mentioned, SVD-based noise reduction was able to improve the precision and recall levels. We obtained a gain of up to 10.3% with the KNN classifier, 35.0% with naive Bayes and 16.2% with random forest. Interestingly, we verified that the different classifiers achieved comparable results after the use of SVD for dimensionality reduction (all levels remained above 90%). Dimension reduction ability is important for scalability in this scenario because many protein domains are experiencing exponential growth. There are about 110,000 domains, i.e., instances to classify, in the SCOP database. Each of these instances can be represented by 151 attributes (dimensions) in the case of the CSM with a cut-off of up to 30Å.

**Figure 2:**
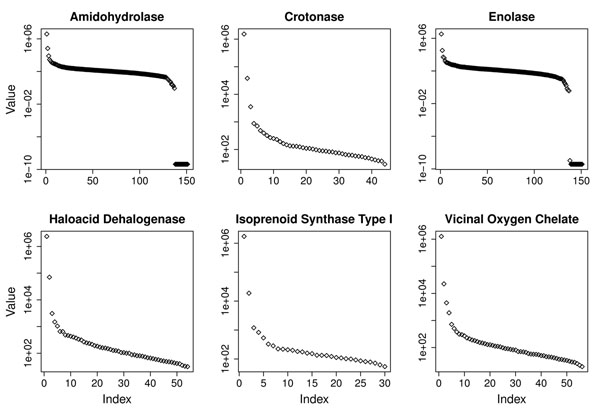
**Singular value distribution.** Singular value distribution obtained after the execution of the SVD routine for each superfamily considered in the gold-standard dataset. A sudden drop in the singular values denotes the cutoff point for dimensionality reduction. The Y-axes have a logarithmic scale.

**Figure 3:**
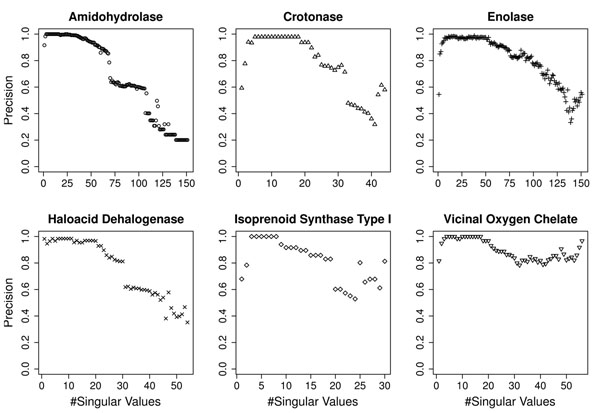
**Influence of the number of singular values chosen in precision.** Influence of the cutoff point for dimensionality reduction in the average weighted precision for the superfamilies considered in the gold-standard dataset. A drop in the precision after a certain number of singular values might indicate the point where noisy components start to appear.

To find the point that maximizes the noise reduction, we studied the singular value distribution obtained for the gold-standard dataset. Figure 2 shows the elbow of the curve of the contribution of each singular value to represent the original information. Using about 9 dimensions we can represent the same information (reducing the noise) and obtain very high precision in classification with a considerably smaller dataset. As shown in Figure 3, maximum precision can be achieved with about 9 singular values for all experiments.

## Conclusions

Function and fold prediction, while means of understanding the composition, operation, interaction and evolution of proteins, are still great challenges in the face of the explosive growth of protein data generation and storage in public databases. To keep up with the frenetic pace imposed by this increasing data availability, novel, efficient methods for automatic and semi-supervised annotation are needed. As a mechanism to exploit the close relationship between protein structure and function, we developed a structure-based method for function prediction and fold recognition based on protein inter-residue distance patterns. The motivation for this approach arose from the hypothesis that proteins with different structures would show different inter-residue distance patterns, and structural similarity would be reflected in these distances.

One of the most remarkable advantages of the CSM-based structural signature is its generality, as we successfully instantiated it in different problem domains, such as function and fold prediction. Also, as a requirement and demand for its application to databases that are continuously growing, it is scalable for real-world scenarios, such as whole-SCOP classification tasks, as shown in previous sections, and it shows an efficacy comparable or superior to state-of-the-art protein folding and function predictors. We would like to stress that our method is probably the first to present a full-SCOP automatic classification in acceptable time (a few hours in a quad-core machine).

The interpretation and understanding of the intrinsic distance patterns generated by CSM demand further investigation. As part of future studies, we intend to explore the generality of CSMs in other aspects of protein function, such as subcellular localization prediction and prediction of GO terms, as well as under different structural classification databases, such as CATH [30]. We also plan to contrast SVD with feature selection as methods for discriminant information discovery in CSMs.

Furthermore, the significant gain in prediction power provided by SVD processing might imply that there is room to improve in terms of the data input, indicating that other cutoff ranges and granularities should also be tested, which is a study already in progress in our group.

## Methods

### CSM-based approach

**Figure 4:**
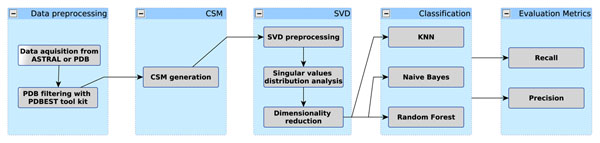
**CSM-based function prediction and fold recognition workflow.** A schematic view of the CSM-based function prediction and fold recognition approach is shown. The workflow is divided into steps of data preprocessing, CSM generation, SVD dimensionality reduction, classification and evaluation.

Figure 4 gives a schematic view of the CSM-based approach for protein function prediction and fold recognition employed in this work, which can be divided into data preprocessing, CSM generation, SVD-based dimensionality reduction and classification steps.

After the data acquisition and filtering steps for a certain dataset (designed either for function prediction or fold recognition purposes), the CSMs are generated (the details of the procedure are explained later in this section). The CSM defines a feature vector that is then processed with SVD. To define a threshold value for dimensionality reduction, the singular values distribution is analyzed. The elbow of this distribution is used as a threshold for data approximation and recomposition (the explanation of the SVD procedure is detailed in the next subsections) and indicates that the contribution of the other singular values to describing the matrix is insignificant, and thus they might be seen as noise.

These singular values are then discarded. Finally, the processed CSM is submitted for classification tasks under different algorithms. Metrics such as precision and recall are calculated to assess the prediction power of the classifiers.

### Cutoff scanning matrices

In a previous work [26], we conductedd a comparative analysis between two classical methodologies to prospect residue contacts in proteins, one based on geometric aspects, and the other based on a distance threshold or cutoff, by varying (scanning) this distance to find a robust and reliable way to define these contacts. In the present work, we used the cutoff scanning approach for classification purposes, which is the basis of the CSMs. The motivation for the use of this kind of information relies on the fact that proteins with different folds and functions present significant differences in the distribution of distances between their residues. On the other hand, one can expect that proteins with similar structures would also have similar distance distributions between their residues, information that is captured in a CSM.

The CSMs were generated as follows: for each protein of the datasets, we generated a feature vector. First, we calculated the Euclidean distance between all pairs of *C_α_* and defined a range of distances (cutoffs) to be considered and a distance step. We scanned through these distances, computing the frequency of pairs of residues, each represented by its *C_α_*, that are close according to this distance threshold. Algorithm 1 shows the function that calculates the CSM.

In this work, we vary the distance threshold from 0.0 Å to 30.0 Å, with a 0.2-Å step, which generates a vector of 151 entries for each protein. Together, these vectors compose the CSM. In short, each line of the matrix represents one protein, and each column represents the frequency of residue pairs within a certain distance. Alternatively, this frequency might be seen as the number of contacts in the protein for a certain cutoff distance or the edge count of the contact graph defined using that distance threshold. This step was implemented in the Perl programming language.

It is important to mentioned that other centroids could be chosen instead of the *C_α_*, such as the *C_β_* or the last heavy atom (LHA) of the side chain. Additional file 1, Figure S3 shows the performance comparison between the *C_α_* and *C_β_* for the EC number dataset. The *C_α_* performed better in all experiments, a fact that demands further investigation.

**Figure 5:**
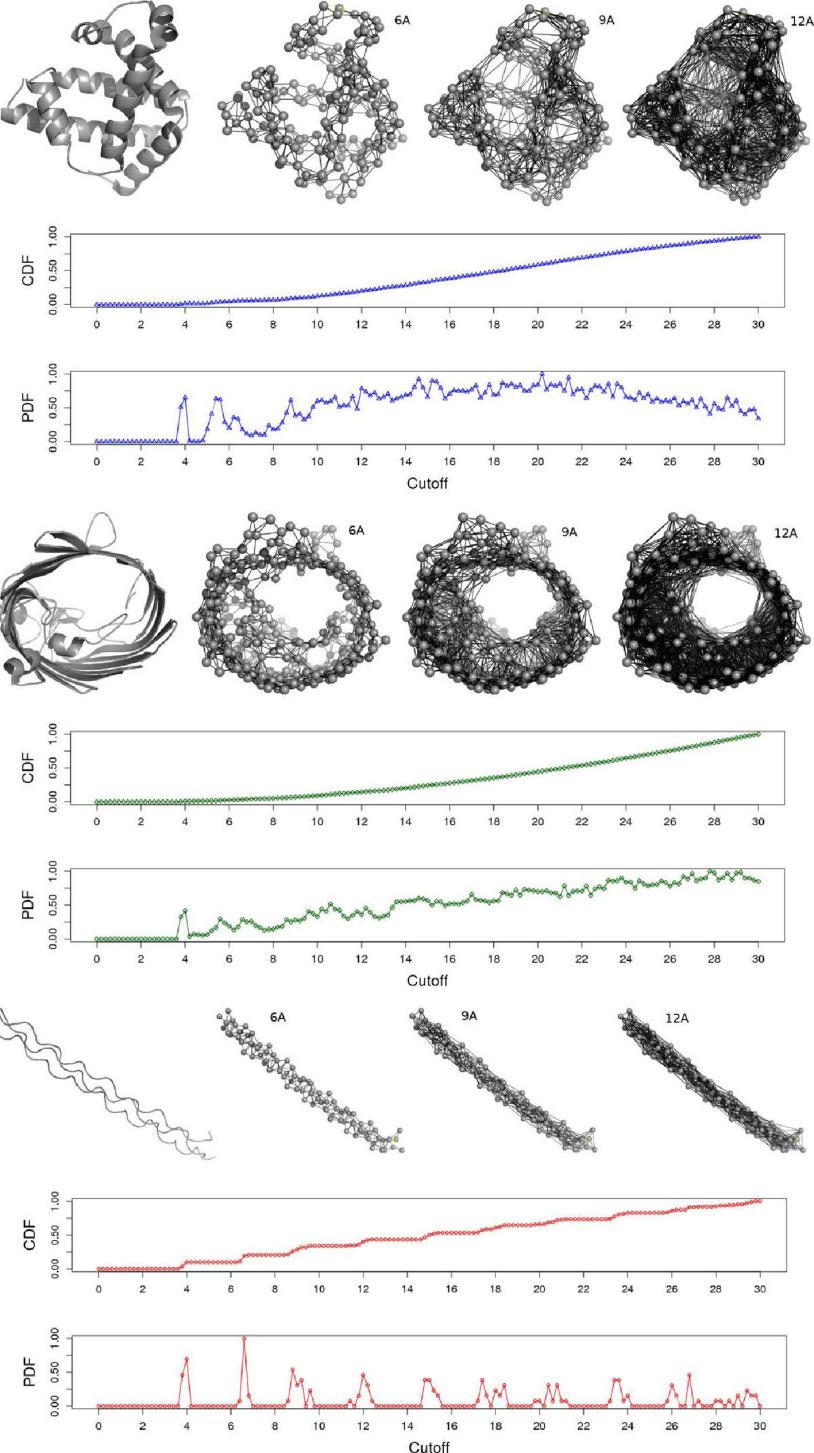
**Contact graphs topology per cutoff for proteins with different folds.** The topologies of the contact graphs of three distinct structures (from top to bottom: globin, porin and collagen) at different cutoff values: 6.0Å, 9.0Å and 12.0Å are shown. The edge count for each graph represents an entry in the cutoff scanning feature vector. The normalized cumulative distribution and density distribution of the cutoff scanning profile of these proteins are also shown.

The motivation for using CSMs comes from the differences in the contact distributions for proteins of different structural classes, as can be seen in Additional file 1, Figure S4, which shows the normalized edge count density distribution per cutoff for proteins from different SCOP classes, namely: *all alpha*, *all beta*, *alpha*+*beta* and *alpha/beta*. It is possible to see that the differences between the distributions emerged at different cutoff ranges. For example, the first peaks for the alpha proteins indicate first-order contacts of their helices and the differences at higher cutoffs might happen due to the diameter and density of the proteins. We stress that these variations in the edge count are not only a phenomenon of the secondary structure composition of the proteins but a phenomenon of the protein packing itself. It is important to explain the cutoff variation. The cutoff variation (scanning) aggregates important information related to the packing of the protein and captures, implicitly, the protein shape. We believe that pockets on the surface and even core cavities are well accounted for by this novel type of structure data we proposed. Another example of contact distributions is shown in Figure 5. Three proteins with very different shapes were selected (a globin, PDB:1A6M; a porin, PDB:2ZFG; and a collagen, PDB:1BKV), and the topology of the contact graph obtained with different cutoffs is shown (6.0 Å, 9.0 Å and 12.0 Å). The cumulative and normalized density distributions for the CSM feature vectors for these representatives are also plotted. We can see from these examples that an expressive difference in shape is accounted for in the CSM. In the contact profile, the peaks indicate high frequency of recurrent distance patterns present in proteins structures. A higher peak under 3.8-4.0 Å provides evidence for the distances given by consecutive *C_α_*s. These distances will tend to be independent of the protein structural class in face of the planar property that characterize the peptide link intermediating two contiguous *C_α_*s in the chain. In addition to this pattern, in proteins rich in helices, we will find new suggestive peaks between 5.0 Å and 7.0 Å, representing mainly the recurrent distances between the local (in sequence) *C_α_*s positions (*i*, *i* + 2), (*i*, *i* + 3) and (*i*, *i* + 4) that compose turns of a helix, and also some nonlocal contacts. Conversely, in proteins rich in beta strands, important peaks will be noted around 6.0 Å and 5.0 Å, referring not only the distances in local *C_α_* positions (*i*, *i* + 2) but also nonlocal *C_α_* contacts (*i*, *i* + *k*) present in companion strands. This implies that CSM is manipulating two essential structural information levels: local and nonlocal relevant contacts. We also can see that the shapes of the proteins directly interfere in the underlying contact network, which is reflected in the protein folding, as pointed by [25]. These properties make the CSM a rich and important source of information when dealing with problems like protein function prediction and structural classification.

### Noise reduction with SVD

To reduce the inherent noise in the generated data and also reduce the cost of the classification algorithms in terms of execution time and memory requirements, we used an SVD-based dimensionality reduction. SVD establishes non-obvious, relevant relationships among clustered elements [31-33]. The rationale behind SVD is that a matrix *A*, composed of *m* rows by *n* columns, can be represented by a set of derived matrices [33] that allows for a numerically different representation of data without loss in semantic meaning. That is:

Where *T* is an orthonormal matrix of dimensions *m* x *m*, *S* is a diagonal matrix of dimensions *m* x *n* and *D* is an orthonormal matrix with dimensions *n* x *n*. The diagonal values of *S* are the singular values of *A*, and they are ordered from the most to the least significant values.

When considering only a subset of singular values of size *k* <*p*, where *p* is the rank of *A*, we can achieve *A_k_*, an approximate matrix of the original matrix *A*:

Thus, data approximation depends on how many singular values are used [34]. In this case, the *k* number of singular values is also the rank of the matrix *A_k_*. The possibility of extraction of information with less data is part of this technique’s success, as it can permit data compression/decompression within a non-exponential execution time, making analysis viable [34]. A dataset represented by a smaller number of singular values than the full-size original dataset has a tendency to group together certain data items that would not be grouped if we used the original dataset [33]. This grouping could explain why clusters derived from SVD can expose non-trivial relationships between the original dataset items [35]. In this paper, we use *A_k_*, the product’s factorization by SVD, to rank *k*, but with only two arrays of SVD, the matrix *V_k_*[32] can be represented in the context of the matrix:

The justification for using only *V_k_* is that the relationships among the columns of *A_k_* are preserved in *V_k_* because *T_k_* is a base for the columns of *A_k_*.

We evaluated the singular values distribution in an effort to find a good threshold to reduce the number of dimensions without losing information. This step, as well as the generation of all graphics, was performed via R programming language scripts.

### Evaluation methodology

An extensive series of experiments was designed to evaluate the efficacy of CSMs as a source of information for protein fold recognition and function prediction.

In the classification tasks, the Weka Toolkit [36], developer version 3.7.2 was used. For the gold-standard dataset, three classification algorithms were used, and their performances were compared: KNN, random forest and naive Bayes. For the other datasets, KNN was used. The algorithms’ parameters, when applicable, were varied and the best result computed. In all scenarios, 10-fold cross validation was applied. The classification performance was evaluated using metrics such as *precision* (*Precision* = *TP/*(*TP* + *FP*)), *recall* (*Recall* = *TP/*(*TP* + *FN*)), *F1 score* (the harmonic mean between precision and recall: ) and the Area Under the ROC Curve (AUC). The variation in precision was used to measure the gain obtained with SVD processing, and the recall variation was evaluated to compare the results with those for the dataset derived from [29].

We also correlated the precision obtained by the classifiers and the number of singular values considered and compared it with the results using the whole CSM.

### Datasets

Our datasets consisted of proteins structures available in the Protein Data Bank [4]. The domains covered by SCOP release 1.75 were obtained through the ASTRAL compendium [37]. The protein structures were grouped according to the purpose of the experiment, namely, function prediction or fold recognition. For structures solved by NMR, we only considered the first model. The chains were split into separate files and the *C_α_* co-ordinates extracted using PDBEST toolkit.

The first dataset concerns a gold-standard of mechanistically diverse enzyme superfamilies [28]. We consider *six superfamilies* (amidohydrolase, crotonase, haloacid dehalogenase, isoprenoid synthase type I and vicinal oxygen chelate), comprising 47 families distributed among 566 different *chains*. The list of PDB IDs as well as the family and superfamily assignments are available in Additional file 2.

The second dataset contains enzymes with EC numbers. We considered the top 950 most-populated EC numbers in terms of available structures, with at least 9 representatives per class, in a total of 55,474 chains, which covered 95% of the reviewed enzymes from Uniprot [1], i.e., the experimentally validated annotations from that database.

The third dataset originated from SCOP version 1.75 for fold recognition tasks. We selected all PDB IDs covered by SCOP with at least 10 residues and 10 representatives per node in the SCOP classification hierarchy. These IDs represented a total of 110,799, 108,332, 106,657 and 102,100 domains at the class, fold, superfamily and family levels, respectively. We would like to emphasize that this is a very large dataset and that we found no other paper relating the use of such a complete dataset in strutcural classification tasks. The last dataset was derived from [29] for comparison in fold recognition tasks. We selected all domains described in its additional files with a minimum of 10 representatives per node in the SCOP classification hierarchy. It was not possible to identify exactly the domains they used from the additional files and only those pairs of domains with a sequence identify below 35% were retained. It is important to stress that the work of Jain and colleagues only contemplate structures with 3, 4, 5 or 6 secondary structure elements.

## List of abbreviations used

EC: Enzyme Commission; CSM: Cutoff Scanning Matrix; DUF: Domain of Unknown Function; SVD: Singular Value Decomposition; PDB: Protein Data Bank; SCOP: Structural Classification of Proteins; GO: Gene Ontology; LHA: Last Heavy Atom; KNN: K-Nearest Neighbors; AUC: Area Under the ROC Curve.

## Competing interests

The authors declare that they have no competing interests.

## Authors' contributions

DEVP conceived of the study, developed the algorithms, performed the experiments and drafted the manuscript. RCMM participated in the design of the study, helped with presenting and analyzing the results and drafted the manuscript. MAS participated in the design of the study, provided advice on the SVD analysis and helped draft the manuscript. CHS helped with presenting the results, provided advice on its analysis and helped draft the manuscript. MMS helped draft the manuscript and provided advice on analyzing the results. WM participated in the coordination of the study and helped draft the manuscript. All authors read and approved the final manuscript.

## Supplementary Material

Additional file 1 **Additional figures and tables.** Figure S1 - Performance metrics across EC classes. Figure S2 - Correlation between precision and minimum number of representatives. Figure S3 - The influence of *C_α_* and *C_β_* distances in the performance. Figure S4 - Feature vector density distribution for proteins of different SCOP classes. Table S1 - Function prediction performance using naive Bayes for gold-standard dataset. Table S2 - Function prediction performance using random forest for the gold-standard dataset. Click here for file

Additional 2 **Enzyme gold-standard dataset.** List of PDB identifiers that compose the enzyme gold-standard dataset and its family and superfamily assignments. Click here for file
