# Supplementary material for: Cutoff Scanning Matrix (CSM): structural classification and function prediction by protein inter-residue distance patterns
Source: BMC Genomics. 2011 Dec 22;12(Suppl 4):S12. doi: 10.1186/1471-2164-12-S4-S12 (PMC3287581; doi:10.1186/1471-2164-12-S4-S12)
Supplement: Additional file 1 — Additional figures and tables. Figure S1 - Performance metrics across EC classes. Figure S2 - Correlation between precision and minimum number of representatives. Figure S3 - The influence of Cα and Cβ distances in the performance. Figure S4 - Feature vector density distribution for proteins of different SCOP classes. Table S1 - Function prediction performance using naive Bayes for gold-standard dataset. Table S2 - Function prediction performance using random forest for the gold-standard dataset. [file 1471-2164-12-S4-S12-S1.pdf]

Cutoff Scanning Matrix (CSM): structural classification and function prediction by protein inter-residue distance patterns

Douglas E V Pires*[1,2], Raquel C de Melo-Minardi[2], Marcos A dos Santos[2], Carlos H da Silveira[3], Marcelo M Santoro[1], Wagner Meira Jr.[2]

[1]Department of Biochemistry and Immunology, Universidade Federal de Minas Gerais, Belo Horizonte, 31270-901, Brazil
[2]Department of Computer Science, Universidade Federal de Minas Gerais, Belo Horizonte, 31270-901, Brazil
[3]Advanced Campus at Itabira, Universidade Federal de Itajubá, Itabira, 37500-903, Brazil

Email: Douglas E V Pires*- dpires@dcc.ufmg.br; Raquel C de Melo-Minardi - raquelcm@dcc.ufmg.br; Marcos A dos Santos - marcos@dcc.ufmg.br; Carlos H da Silveira - carlos.silveira@unifei.edu.br; Marcelo M Santoro - santoro@icb.ufmg.br; Wagner Meira Jr. - meira@dcc.ufmg.br;

*Corresponding author

Additional figures and tables

Figure S1 - Performance metrics across EC classes

The variation in the performance metrics for each EC number class is shown. The majority of the EC numbers was well classified according to the metrics shown.

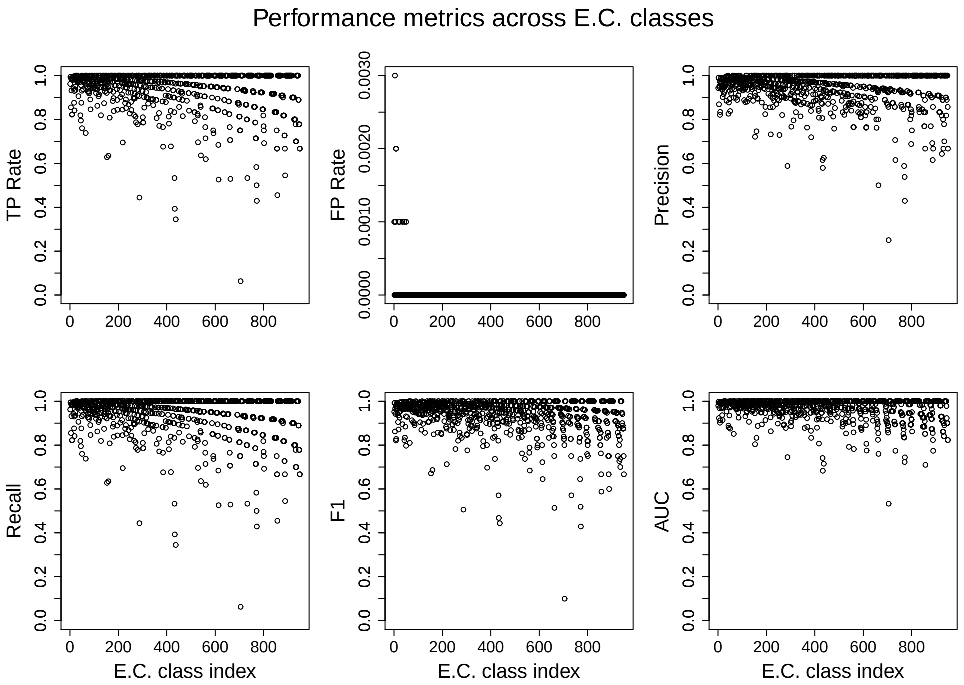

Figure S2 - Correlation between precision and minimum number of representatives

Correlation between the average weighted precision for the full-SCOP dataset, with and without the execution of SVD, and the minimum number of entities per *class* in the classification task. In this context, *class* should be understood as the group of entities with the same SCOP classification for a certain classification level: fold, superfamily or family in this case.

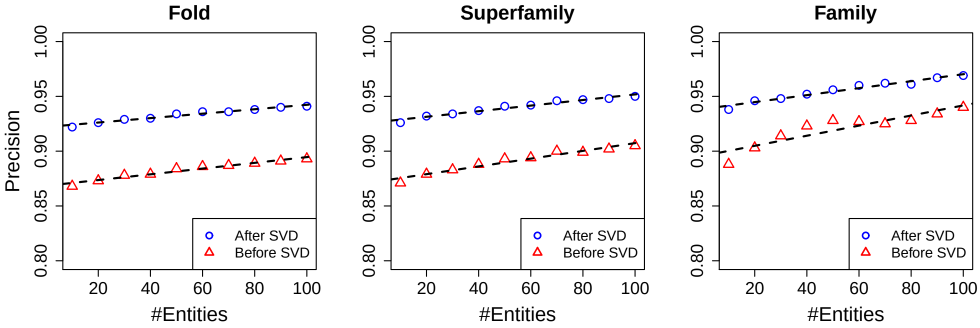

Figure S3 - The influence of C_α and C_β distances in the performance

The comparative performance for the EC number dataset when using the C_α or C_β distances to generate the Cutoff Scanning Matrix is shown. In all experiments, the alpha carbon presented a better performance in terms of the metrics presented in the figure.

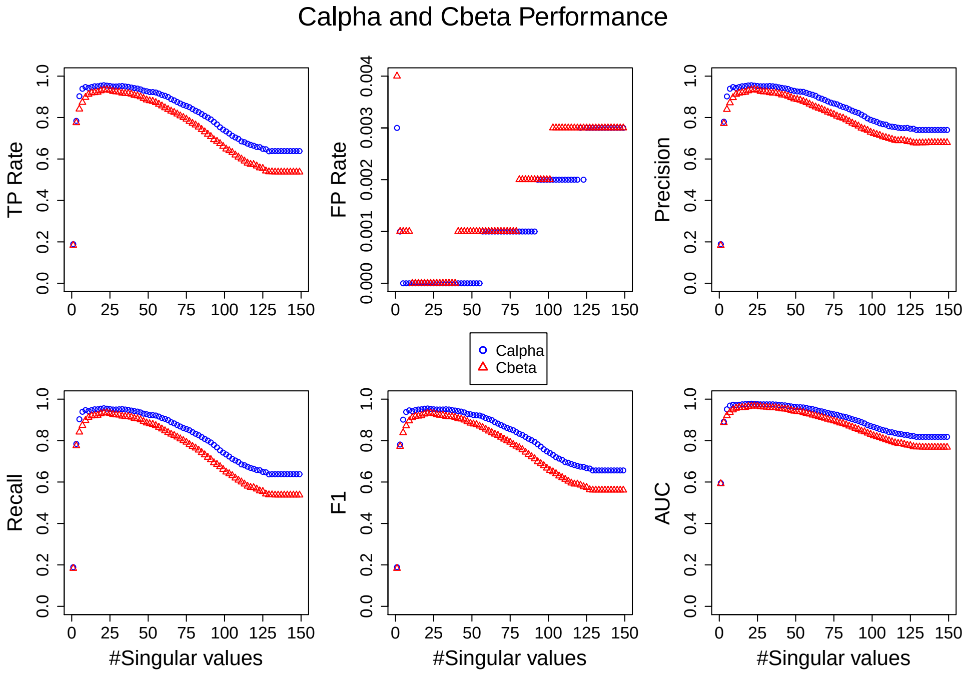

Figure S4 - Feature vector density distribution for proteins of different SCOP classes
Density distribution for cutoff scanning feature vectors for proteins of the first four SCOP classes. Each curve represents the mean values of ten randomly selected representatives per class.

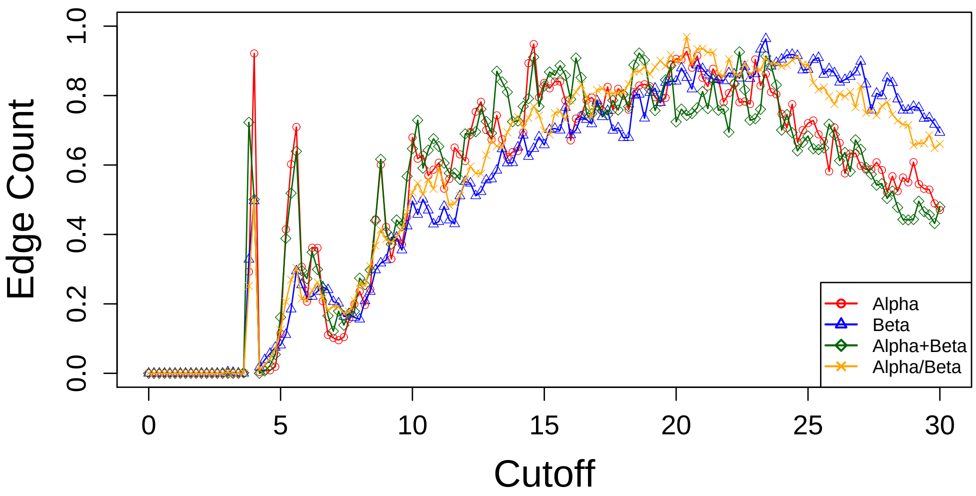

Table S1 - Function prediction performance using naive Bayes for gold-standard dataset
Prediction performance for the gold-standard dataset using naive Bayes. The experiment was performed in an intra-superfamily fashion, and the *classes* for prediction represent the enzyme's families. The precision and recall metrics are weighted averages. 10-fold cross validation was employed.

**Superfamily**	**Before SVD**		**After SVD**		**ΔPrec.**	**ΔRec.**
	Precision	*Recall*	*Precision*	*Recall*		
Amidohydrolase	0.985	0.983	1.000	1.000	+1.5%	+1.7%
Crotonase	0.754	0.698	0.979	0.977	+22.5%	+27.9%
Enolase	0.596	0.580	0.946	0.931	+35.0%	+35.1%
Haloacid Dehalogenase	0.863	0.830	0.971	0.962	+10.8%	+13.2%
Isoprenoid Synthase Type I	0.970	0.966	0.970	0.966	+0.0%	+0.0%
Vicinal Oxygen Chelate	0.855	0.836	0.983	0.982	+12.8%	+14.6%
All	0.741	0.655	0.946	0.933	+20.5%	+27.8%

Table S2 - Function prediction performance using random forest for the gold-standard dataset
Prediction performance for the gold-standard dataset using random forest. The experiment was performed in an intra-superfamily fashion, and the *classes* for prediction represent the enzyme's families. The precision and recall metrics are weighted averages. A 10-fold cross validation was employed.

**Superfamily**	**Before SVD**		**After SVD**		**ΔPrec.**	**ΔRec.**
	Precision	*Recall*	*Precision*	*Recall*		
Amidohydrolase	0.983	0.983	0.996	0.996	+1.3%	+1.3%
Crotonase	0.844	0.837	0.979	0.977	+13.5%	+14.0%
Enolase	0.815	0.807	0.977	0.973	+16.2%	+16.6%
Haloacid Dehalogenase	0.982	0.981	0.986	0.981	+0.4%	+0.0%
Isoprenoid Synthase Type I	0.970	0.966	1.000	1.000	+3.0%	+3.4%
Vicinal Oxygen Chelate	0.947	0.945	0.984	0.982	+3.7%	+3.7%
All	0.898	0.892	0.991	0.991	+9.4%	+9.9%
